# Histopathological findings in a mass mortality event in a *Helix aspersa maxima* farm*:* possible association with calcium metabolism alterations

**DOI:** 10.1016/j.vas.2026.100682

**Published:** 2026-05-06

**Authors:** Rebecca Leandri, Gionata De Vico, Alberico Franzin, Carla Ferraris, Karen Power

**Affiliations:** a Dipartimento di Biologia, Università degli Studi di Napoli Federico II, Via Vicinale Cupa Cinthia 26, 80126, Naples, Italy; b Istituto Zooprofilattico Sperimentale del Piemonte, Liguria e Valle d’Aosta, Via Bologna 148, 10154, Turin, Italy

**Keywords:** Calcium, Heliciculture, Mortality, Calcification, Snails

## Abstract

Heliciculture is becoming an interesting sustainable opportunity to increase farming yields, however effective good farming practices are lacking. Following a mass mortality event in a snail farm, samples of *Helix aspersa maxima* were analyzed with anatomo-histopathological techniques.

Macroscopic examination revealed alterations of the gastro-enteric apparatus and of the digestive gland, which at the microscopic examination showed dysregulation in the assembly of calcium. Also, the salivary glands and the albumen gland presented different degrees of alterations ranging from atrophy to dystrophy. Interestingly, alterations of calcium localization and mineralization were observed in different organs.

Our histopathological results suggest the possible implication of calcium metabolism dysregulation in contributing to the mortality events here described. Incorrect feeding and management practices, heavy metal intoxication/detoxification mechanisms, and chronic heat stress should all be considered as possible causes and further investigations focused on the pathological effects of management and environmental triggers in snail productions should be carried out to fill the gaps of knowledge in this field.

## Highlights

• Mass mortality in Helix aspersa maxima linked to calcium overfeeding.
• Severe alterations found in digestive, salivary, and albumen glands.
• Calcium cells showed abnormal mineralization and necrotic processes.
• Calcified aggregates obstructed hemolymphatic vessels in affected snails.
• Findings support the role of calcium metabolism dysregulation in mortality.

## Graphical abstract

**Figure:**
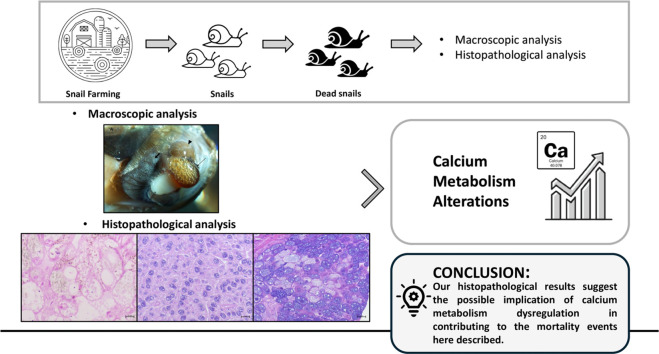

## 1 Introduction

Snails belong to the phylum Mollusca and the class of gastropods, to which about 1900 of species pertain (Appukuttan & Ramadoss, 2000; Bouchet et al., 2005, 2017).

Among those species, some have destructive effects on plants and are identified as agricultural pests, while others such as *Helix aspersa, Helix pomatia, Archachatina marginata, Achatina achatina, Achatina fulica* and *Helix lucorum* are of nutritional, aesthetic, medical or veterinary interest (Adegoke et al., 2010; Cilia & Fratini, 2018; Singh et al., 2024). Snails have been part of human nutrition since the Late Pleistocene and Early Holocene periods and they remain part of the traditional cuisines of numerous European nations (Elmslie, 2005; Miracle et al., 2000) and as a result, the demand for snail farming is valuable and is expected to grow, particularly in Mediterranean regions (https://www.indexbox.io/store/world-snails-except-sea-snails-market-analysis-forecast-size-trends-and-insights/ accessed on 13April 2026). Indeed, snail caviar and meat are consumed for their high quality nutritional profile (Adegoke et al., 2010; Gupta & Khanal, 2024) and farming and consumption of snails has increased in the past years (Rygało-Galewska et al., 2022). The nutritional compositions can vary according to the species, generally containing great amounts of crude proteins (∼8.3–20.6 %) which are comparable with conventional meats such as Beef (20.6 %), Pork (22.8 %), Chicken (19.3 %) and Fish (19.8 %) (Bender, 1992). As all meat sources, snail meat contains all essential amino acids as well as fatty acids, macro and microminerals (Ghosh et al., 2022).

In recent decades, heliciculture has changed from traditional backyard practices to a structured agricultural industry, particularly in European countries like Spain, Portugal, Italy, France, and Greece where it has been popular from historical traditions (Dragicevic & Baltic, 2005; Miracle et al., 2000). In France alone, annual consumption exceeds 30,000 tons, much of which is derived from farming systems based primarily on *Helix aspersa* (now often referred to as *Cornu aspersum*) (Elmslie, 1989). Italy has similarly seen rapid growth (Zucaro et al., 2016), with structured production models such as the Italian outdoor system promoting standardization of breeding, management and productions (https://www.istitutodielicicoltura.com/en/the-method/ accessed on 15 May 2025). Also, African countries like Nigeria, Ghana, and Morocco are strong consumers of snail meat, and snail farming becomes a way to fight poverty and malnutrition (Agbogidi & Okonta, 2011; Adeyeye et al., 2020;).

As a matter of fact, edible snail farming can become an interesting opportunity to increase farming yields as snails do not require initial great financial investments, nor human labour, they require relatively little space for breeding, and they have good conversion parameters (Hatziioannou et al., 2014; Murphy, 2001). Furthermore, heliciculture is considered as a sustainable practice under both the economic and environmental point of view (Zucaro et al., 2016). A study by Forte et al., (2016) estimated the carbon footprint of heliciculture, and results showed that it amounted to 0.7 kg CO2 eq per kg of edible meat, mainly ascribable to supplementary feeding and to the associated transport. Therefore, heliciculture could raise new sustainable opportunities for rural development of abandoned lands (Mvodo et al., 2021).

Snails can feed on different species of plants, fruits and their discards, as well as vegetable wastes, as long as they are rich in minerals like calcium, essential for the development of snail shells (Gupta & Khanal, 2024; Obakanurhe et al., 2024). It appears that snails have a huge potential in transforming pastures and organic waste into useful nutrient sources (Raimi & Olomola, 2020; Ndu et al., 2023), however when fed with commercial feed the growth rate and the quality of meat appears higher (Garcia et al., 2006; Srijad et al., 2023).

Three different farming systems have been described: internal/intensive, external/extensive mixed/semi-intensive (Apostolou et al., 2021; Cobbinah et al., 2008).

In the first type of breeding system, the farmer can set and manage rearing parameters like temperature, humidity, ventilation and light hours, adapting them to specific needs. This system requires higher investments to create and adequate infrastructure but enables breeding throughout the year despite unfavorable weather conditions, therefore it is a system mainly used in countries with cold winters.

Conversely, in countries with warm climate throughout the year, external systems are more frequent. In these systems, animals are raised in plots throughout all the farming stages from development to overwintering. While initial investment in external farming is lower, the siting of the farming site must be chosen carefully to avoid chemical contamination of environment, predation, unfavorable soil quality and weather. The most common snail farming system is the mixed one, in which breeding and rearing of juveniles occurs in indoor conditions, while fattening is performed in open airs. This system could help reducing juvenile mortality caused by pests and weather hazards (Rygało-Galewska et al., 2022). Mortality rates are higher in external systems compared to internal systems, as rearing conditions and contact with external elements, such as adverse environmental conditions, can be less controlled (Diederich & Pechenik, 2013; Rygało-Galewska et al., 2022). Despite advances in farming practices, heliciculture continues to face challenges from biological, nutritional, and environmental stressors. Indeed, many studies have reported the association between spawning and mortality of matured snails, suggesting that mortality was higher in individuals presenting higher reproductive efforts which could lead to physical exhaustion and reduced immune capacity (Baur & Baur, 2000; Barker, 2001; de Carvalho et al., 2008; Der et al., 2019). Pathogenic agents, including gram-negative bacteria such as *Vibrio* spp., are often implicated in soft tissue necrosis, shell erosion, and sudden mortality events (Power et al., 2024; Richards-Rios et al., 2024; Segade et al., 2013). Protozoan and helminthic parasites, such as *Angiostrongylus cantonensis, Brachylaima* spp., and *Aelurostrongylus abstrusus* (in intermediate stages), have also been reported and may cause zoonotic or interspecies health risks (De Vico et al., 2017; Godan, 1983). Non-infectious diseases are equally relevant: imbalances in calcium and phosphorus intake can lead to shell deformities, softening, impaired growth, or even metabolic dysfunctions, especially during reproductive phases or environmental stress (Barker, 2001; Garcia et al., 2006; Obakanurhe et al., 2024).

In recent years, unexplained mass mortality events have been increasingly reported in snail farms, particularly during warmer seasons or post-reproductive periods. These events are often characterized by high rates of tissue degeneration, behavioral changes (prolonged inactivity, incomplete shell closure), probably caused by biological agents (De Vico et al., 2017; Power et al., 2024; Richards-Rios et al., 2024; Segade et al., 2013). Nevertheless, histopathological lesions affecting digestive and reproductive organs remain poorly characterized (De Vico et al., 2017; Der et al., 2019). For this reason, there is a growing need to identify pathological patterns and correlate them with potential metabolic or nutritional imbalances. In this context, our study aims to describe the histopathological findings observed in a mass mortality event that occurred in a *Helix aspersa maxima* farm in Southern Italy and we here suggest that observed lesions could be associated to calcium metabolism dysregulation.

## 2 Materials and methods

In September 2024 a snail breeder in the province of Salerno, Southern Italy, reported sudden mass mortality of animals raised in his farm.

The snail farm was based on the outdoor snail farming system, and developed across a 5000 m^2^ surface with a pasture made of Brassicaceae.

Animals were purchased from a retailer as baby snail packs and raised in outdoor pens and fed commercial feed (Table 1) provided by the seller following his recommendations. However, as shared by the snail breeder, since the second week of August, extra calcium powder in the form of calcium carbonate was provided every two days until the first week of September. Unfortunately, it was not possible to take samples of the calcium matrix.

**Table 1:** Nutritional sheet of the commercial feed administered.

Analytical Components	Percentage (in 100 g)
Crude protein	12.6%
Crude fat	2.6%
Crude fiber	7.0%
Crude ash	24.0%
Calcium	8.0%
Magnesium	0.2%
Sodium	0.0%

### 2.1 Sampling

One hundred samples of moribund *Helix aspersa maxima* were randomly collected from the farm pens while ten samples of healthy *H. a. maxima* were collected from outside the pens in the surrounding area. Considering that the latter were not managed and naturally pastured on the available plants, they were used as controls. All samples were immediately transported in nets to the Department of Biology, University of Naples Federico II for macroscopic and microscopic examination.

### 2.2 Macroscopic and microscopic analysis

Macroscopic examination was performed on all organs after shell removal. Samples were fixed in Davidson solution for 72 h and then processed for histologic examination as previously described (Power et al., 2024). 3 μm thick sections were stained with hematoxylin and eosin (h&e) and observed by light microscopy to identify possible tissue alterations; moreover, samples showing tissue lesions were further cut, and sections were stained with von Kossa staining to identify calcium accumulation in tissues.

## 3 Results

### 3.1 Gross pathology

The shell of moribund samples of *H. a. maxima*, presented a shallow groove.

After shell removal samples presented alterations of the gastro-enteric apparatus which appeared swollen and engorged with bubbly red liquid, disarranging the normal anatomy of the genital system and the digestive gland (DG).

**Fig. 1:**
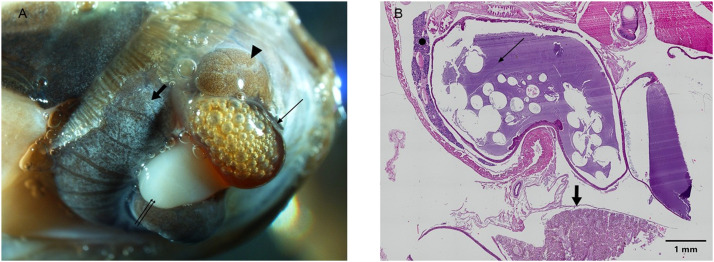
*H. a. maxima*. (A) Gross pathology. Swollen stomach with bubbly red liquid (thin arrow), genital system (double arrow) and the digestive gland (arrow head) with white grains (fat arrow) are showed. (B) Histopathology. Basophilic material and air in the stomach (thin arrow). Microscopic lesions in the digestive gland (fat arrow) and the salivary glands (dot) (h&e stain 4x, scale bar: 1 mm).

The DG was opaque and dark brown to black, often intermingled by white “grains” (Fig. 1 A-B). Some samples evacuated white, grainy feces during manipulation.

Macroscopic observation of control samples did not reveal any alterations of the DG nor of other organs.

### 3.2 Histopathology

Histopathological examination revealed lesions in several organs, namely the DG, the salivary glands (SG), and the albumen gland (AG).

**Fig. 2:**
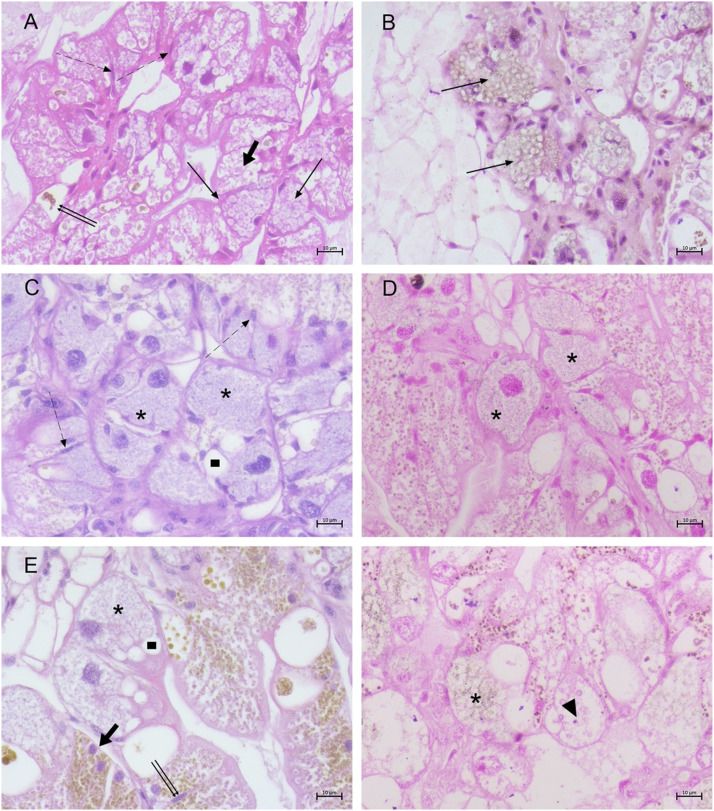
*H. a. maxima*: digestive gland of control (A-B) and of moribund (C-F) animals. (A) Epithelium with thin cells (dashed arrows), digestive cell (fat arrow), calcium cells (thin arrows), excretory cells (double thin arrows) (h&e stain 40 ×, scale bar: 10 µm); (B) Calcium cells with calcium spherules (thin arrows) (von Kossa stain 40x, scale bar: 10 µm); (C) Pulverulent deposits (asterisks) and vacuoles in calcium cells (rectangle), and thin cells (dashed arrows) (h&e stain, 40 ×, scale bar: 10 µm); (D) Pulverulent deposits in calcium cells (von Kossa stain, 40 ×, scale bar: 10 µm); (E) Calcium spherules (asterisks) in vacuolated calcium cells (rectangle). Thin cells (double thin arrows) and digestive cells (thick arrow) (h&e stain, 40 ×, scale bar: 10 µm); (F) Calcified spherules (asterisks) and uncalcified ones (arrowhead) in calcium cells (von Kossa stain, 40 ×, scale bar: 10 µm).

**Fig. 3:**
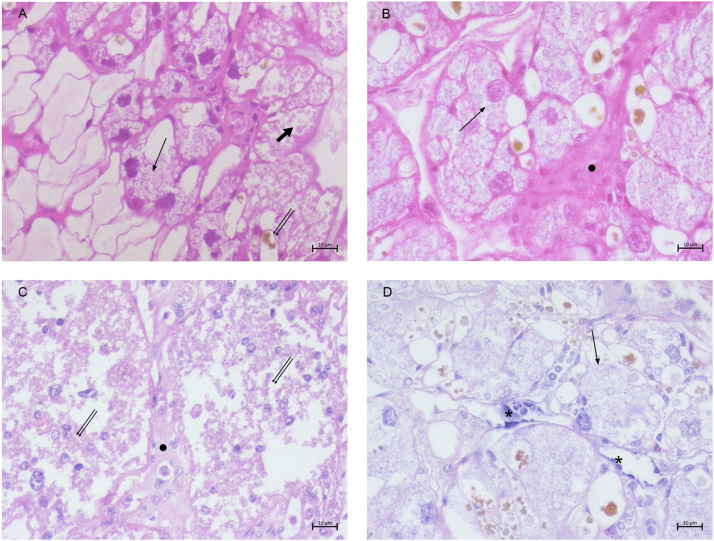
*H. a. maxima*: digestive gland of control (A) and moribund animals (B-D). (A) Epithelium with digestive cell (fat arrow), calcium cells (thin arrows), excretory cells (double thin arrows) (h&e stain 40 ×, scale bar: 10 µm); (B) Hypertrophic and hyperplastic calcium cells (thin arrow) and interstitial fibrosis (dot) (h&e stain, 20 ×, scale bar: 20 µm); (C) Necrotic areas (double thin arrows) with interstitial fibrosis (dot) (h&e stain, 40 ×, scale bar: 10 µm); (D) Calcium cells (thin arrow) and basophilic precipitates in the lumen and along vessel walls (asterisk).(h&e stain, 40x, scale bar: 10 µm).

The DG of control samples presented an epithelium composed of 4 cell types (thin cells, digestive cells, calcium cells, and excretory cells) lying on a thin basal membrane (Fig. 2 A and Fig. 3 A).

In accordance with literature (Wagge, 1951) calcium was stored within small spherule located near the nucleus and, when abundant, filling the whole cytoplasm of calcium cells (Fig. 2 B).

In the 82% (82/100) of moribund animals, the DG showed hypertrophy and hyperplasia of the calcium cells, delimited by thickened basal membrane (Fig. 3 B). In the severest cases (20/82, 24%) necrosis associated with inflammation and interstitial fibrosis (Fig. 3 C) was observed together with hyperplasia of the thin cells, suggesting attempted regeneration of the tissue.

Frequently (30/82, 36.6%), the cytoplasm of the calcium cells presented hemocytes and various vacuolations localized at the cell apex in the context of dispersed eosinophilic material. Moreover, the presence of basophilic, amorphous material was observed in 40% of affected animals (33/82). This material was found positive to von Kossa staining, confirming it was calcium. An alteration of mineral calcium aggregates was observed in 48% (40/82), often presenting a pulverulent, finely dispersed aspect intermingled to eosinophilic material (Fig. 2 C-D) (13/40, 32.5%,), most frequently attempting to form organized calcified spherule, in the majority of cases resulting in uncalcified ones (Fig. 2 E-F) (27/40, 67.5%). Moreover, in the hemolymphatic vessels of the DG, basophilic precipitates were evident in the lumen and along the walls of the vessel in 22% (18/82) of cases, presumably representing calcified plaques, which obstructed the vessels (Fig. 3 D).

**Fig. 4:**
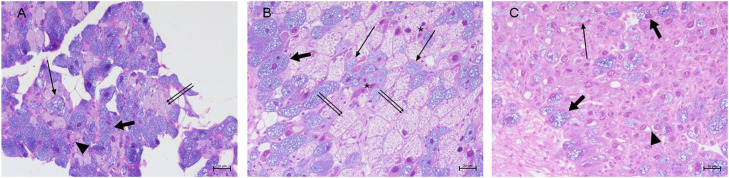
*H. a. maxima*: salivary gland of control (A) and moribund animals (B-C). (A) Mucocytes (thin arrow), large vacuolated cells (double thin arrows), granular cells (thick arrow), cystic cells (arrowhead) (h&e stain, 40 ×, scale bar: 10 µ); (B) immature precursor cells (asterisks), increased vacuolated cells (double thin arrows) and mucocytes (arrow), reduced granular cells (thick arrow) (h&e stain, 20 ×, scale bar: 20 µm); (C) atrophic granular cells (thick arrow), mucocytes (arrow), and empty cystic cells (arrowhead) (h&e stain, 20 ×, scale bar:20 µm).

The SG of control samples consisted of numerous acini surrounding an intralobular duct. Different cell types could be observed: many mucocytes filled with secretory vesicles, numerous large vacuolated cells with oval nuclei and foam-like cytoplasm, granular cells presenting numerous dark granules, and cystic cell characterized by a single large secretory vacuole surrounded by a thin peripheral layer of cytoplasm (Fig. 4 A)

The SG structure of moribund animals was strongly modified in 58% (58/100) of cases, particularly in the number and in the cell types with the ratio of cytotypes altered compared to normal. The presence of numerous undifferentiated immature precursor cells, an increase in vacuolar cells and decrease in granular cells, and limited or absent of cystic cells was noted (Fig. 4 B). Overall, different degrees of alterations ranging from atrophia (31/58, 53.5%) to dystrophia (27/58, 46.5%) of the SG were observed (Fig. 4 C).

**Fig. 5:**
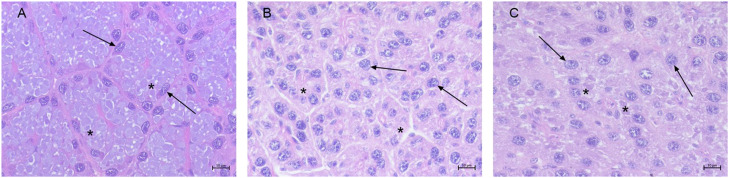
*H. a. maxima*: albumen gland of control (A) and moribund animals (B-C). (A) pyramidal cells (arrow) with basophilic cytoplasmic vacuoles (asterisks) (h&e stain, 40 ×, scale bar: 10 µm); (B) pyramidal cells with granular and eosinophilic cytoplasm (asterisks) (h&e stain, 40 ×, scale bar: 10 µm); (C) cells with basophilic pleomorphic lamellar bodies (asterisks) (h&e stain, 40 ×, scale bar: 10 µm).

The AG of control samples was composed of secretory tubules presenting a reduced lumen, lined with pyramidal cells, with spherical and basal nuclei and basophilic cytoplasmic vacuoles (Fig. 5 A). The AG of moribund samples showed different degrees of structural alterations (53/100, 53%) ranging from atrophia to dystrophia coupled to changes in the quality of secretions: in atrophic AG cytoplasm appeared granular and eosinophilic (Fig. 5 B) (20/53, 38%), while in dystrophic cases basophilic material was found within vacuoles in the form of spherules and pleomorphic lamellar-bodies (Fig. 5 C) (25/53, 47%). In the severest cases hyperchromic nuclei and membrane disruption ending in cell necrosis was observed (8/53, 15%).

Histological examination of the examined samples did not show the presence of parasites, bacteria or viruses nor lesions suggesting their presence.

## 4 Discussion

Mass mortality events in snail farming systems are becoming of growing interest among researchers. Previous studies have ascribed such phenomena to biological agents ranging from viruses, to bacteria, to parasites.

Our study documents systemic regressive tissue alterations (atrophy/dystrophy/necrosis), only rarely found in literature, and for some aspects here described for the first time, in association with a mass mortality event in an intensive snail farm. Of particular importance appear the alterations documented in the DG in relation to the morphology and distribution of the mineralized spherules in the calcium cells, which in our opinion testify to a profound alteration of calcium metabolism in the subjects studied. The calcium cells, in fact, expressed a wide range of morphological alterations whose important functional implications can be deduced from the scientific literature. Calcium cells represent one of the four compartments involved in calcium metabolism and circulation, together with the shell, the connective tissue, and the hemolymph. Calcium cells in pulmonated gastropods are involved in a wide range of metabolic and reproductive functions such as osmoregulation, acid-base balance, formation of the calcium composition of some glandular secretions, and regulation of calcium concentration in the hemolymph (Dittbrenner et al., 2009). Moreover, they represent a storage site for calcium available for shell regeneration and eggshell formation (Fournié & Chétail, 1984; Tompa & Wilbur, 1977). Therefore, it appears clear that alterations of these cells can strongly impair individual health.

Unfortunately, we could not find in literature studies describing similar pathological conditions in pulmonated gastropods, thus we can only make suggestions regarding the pathogenesis of the observed lesions. Different hypotheses can be suggested in reference to incorrect feeding practices, systemic detoxification processes, and chronic heat exposure.

Generally, in healthy snails calcium is stored in the calcium cells as mineralized spherules or concentrically-structured deposits of calcium salts localized at the basal region of the cells (Abolins-Krogis, 1970; Simkiss, 1976). In our samples, we observed alterations in the formation of calcium aggregates and accumulation of pulverulent calcium intermingled to eosinophilic finely dispersed material (likely of proteinaceous origin). It is known that in snails, calcium accumulation and mineralization is strictly connected to calcium and protein dietary intake. Diets rich in calcium and proteins favor the creation in calcium cell of numerous protein spherules enriched with amorphous calcium carbonate. If diet is rich in proteins but lacks calcium, a great amount of protein spherules will be generated but with an 'empty' appearance; if, on the other hand, the diet is rich in calcium but lacks proteins, the calcium cells will contain no calcium aggregates, as carbonates can be easily dissolved in alkaline environments (Wagge, 1951). Taking into consideration the formulation of the commercial diet provided, no strong unbalance between proteins and calcium is evident, however we cannot exclude that there could be other elements triggering physiological calcium metabolism (i.e. calcium antagonists or heavy metals). Nevertheless, the presence of calcium aggregates in different organs, and particularly in the hemolymphatic vessels of the DG, due to possible metastatic calcification, suggests an increase in calcium concentration in the hemolymph which could cause precipitation of calcium carbonate (Greenaway, 1971). Unfortunately it was not possible in that occasion to sample the hemolymph and evaluate the levels of circulating calcium which could undoubtfully confirm hypercalcemia.

Cell calcium overload is recognized as excessive amount of calcium in the cell and has long been associated with disruption of metabolic processes and onset of degenerative and necrotic phenomena, often generated by oxidative stress (Görlach et al., 2015; Peng & Jou, 2010). Recently, calcium overdosing has been associated to the so called “calcicoptosis”, a form of cell death generated from Ca^2+^ accumulation in the cytosol and subsequently in the mitochondria (Zhang et al., 2019). In snails, although calcium represents one of the most limiting elements for growth, development and reproduction (Binh & Thu Thao, 2019; Emelue & Omonzogbe, 2018; Fournié & Chétail, 1984; Gouveia et al., 2011; Rygało-Galewska et al., 2023), the intracellular activity of free calcium must be kept under control, since this element can exert cytotoxic effects at elevated concentrations (Taylor et al., 1988).

In order to avoid calcium intoxication, excess calcium is excreted through feces (Ireland & Marigomez, 1992) or, as a preventive measure, it is sized by a process of biomineralization within small intracellular granules, (Brown, 1982) thus creating an energetically convenient and inactivated stored pool (Beeby, 1991; Howard et al., 1981). This hypothesis has been supported by previous studies in other species, further suggesting that calcium precipitation in subcellular compartments could be and effective mechanism to prevent an excessive concentration of free Ca 2+ in the cytosol, which would be toxic (Bosabalidis, 1987; Davis et al., 1987). The presence of calcic feces and pulverulent calcium in the DG could support the hypothesis of a failed attempt to prevent the effects of calcium overload.

The hypothesis of incorrect feeding could also be supported by the change in the number and type of cells observed in the SG. Healthy SG show a balanced ratio of mucocytes, vacuolated cells, granular cells and cystic cells as previously described (Pirger et al., 2004), while SG of moribund snails showed altered ratio of the different cytotypes and the presence of numerous undifferentiated cells and vacuolar cells, suggesting a selection of some cytotypes probably due to an adaptation to a specific diet, as it occurs in other organs (Lobo-da-Cunha, 2019; Neilson & Terry, 1906; van Weel, 1959).

It is also known that calcium is a relevant element in the regulation of excretory product of the albumen gland: the alteration encountered in the morphology of the AG cells, could well be related to an altered calcium metabolism in the moribund individuals. AG is a female accessory sex gland with a key role in the production of perivitelline fluid coating the fertilized egg (Nieland & Goudsmit, 1969).

This said, we cannot exclude that the calcium unstructured granules could also be regarded as an attempt to detoxify the cell for trace elements (Almedros & Porcel, 1992; Simkiss, 1983, 1981; Simkiss et al., 1982). Previous studies have shown that the calcium granules of the calcium cells are created in strict connection to the Golgi apparatus (Simkiss and Mason, 1983) and it seems that these granules can be released into the lumen of the DG (Mason & Simkiss, 1982), providing a route of trace element excretion. Currently, considering the localization of the snail farm, the farming system and the results of soil analysis (data not shown for privacy reasons), we feel confident in excluding trace elements intoxication as a cause of this particular mass mortality, however it should not be excluded in other mass mortality events occurring in snail farms.

Further hypothesis should consider the influence of chronic heat on the wellbeing of individuals. Indeed, during the summer 2024 Southern Italy experienced unusual heat waves (Benedetti, 2025) which could have activated survival mechanisms in the DG and particularly the calcium cells, which are involved in osmoregulation. Previous studies have described hypertropia and hyperplasia of calcium cells as an attempt to regulate thermal stress and replace heat-damaged cells (Dittbrenner et al., 2009; Scheil et al., 2011) and a concomitant loss of digestive cells, as similarly observed in our study. Although the capability of fighting thermal stress through those mechanism appears to be species-specific, this hypothesis should not be excluded and thermal tolerance should be further investigated also in this subspecies.

## 5 Conclusion

Taken together, our results suggest the hypothesis that an alteration of calcium metabolism could have contributed to the mortality events here described. However, it was not possible to identify an ultimate cause of this impairment as different hypothesis should be taken into consideration. Incorrect feeding and management practices, heavy metal intoxication/detoxification mechanisms, and chronic heat stress should all be considered as possible causes as the digestive gland can be directly affected by those stressors. Sudden events of mass mortality not always provide the possibility of proceeding to investigation following standard procedures, especially when little literature is available for the affected species, thus the need to establish shared protocols.

Our study could open the road to new fields of investigation focused on the pathological effects of management and environmental triggers in minor animal productions, filling the gaps of knowledge in this field.

## Institutional review board statement

Ethical review and approval were waived for this study, as according to the D.L. 4 March 2014 n.26, and the national implementing decree following the European regulation 2010/63/UE, ethical approval is not necessary for invertebrates with the except of cephalopoda**.**

## Ethical statement

Ethical review and approval were waived for this study, as according to the D.L. 4 March 2014 n.26, and national implementing decree following the European regulation 2010/63/UE, ethical approval is not necessary for invertebrates with the except of cephalopoda.

## CRediT authorship contribution statement

**Rebecca Leandri:** Writing – original draft, Visualization, Methodology, Investigation, Formal analysis, Data curation. **Gionata De Vico:** Writing – review & editing, Resources, Conceptualization. **Alberico Franzin:** Methodology, Investigation, Formal analysis, Data curation. **Carla Ferraris:** Methodology, Investigation, Formal analysis, Data curation. **Karen Power:** Writing – review & editing, Supervision, Methodology, Conceptualization.

## Declaration of competing interest

The authors declare that they have no known competing financial interests or personal relationships that could have appeared to influence the work reported in this paper.
